# Impact of antivenom administration on the evolution of cutaneous lesions in loxoscelism: A prospective observational study

**DOI:** 10.1371/journal.pntd.0010842

**Published:** 2022-10-14

**Authors:** Ceila M. S. Malaque, Christina T. G. Novaes, Roberta O. Piorelli, Jose Y. Risk, Jefferson C. Murad, Amanda N. Lara, Cristiana C. Virgulino, Karina T. Miyaji, Marcelo L. Santoro

**Affiliations:** 1 Hospital Vital Brazil, Instituto Butantan, São Paulo, Brazil; 2 Departamento de Moléstias Infecciosas e Parasitárias, Hospital das Clínicas da Faculdade de Medicina da Universidade de São Paulo, São Paulo, Brazil; 3 Laboratório de Fisiopatologia, Instituto Butantan, São Paulo, Brazil; 4 Escola Superior do Instituto Butantan (ESIB), Instituto Butantan, São Paulo, Brazil; Fundação de Medicina Tropical Doutor Heitor Vieira Dourado, BRAZIL

## Abstract

**Background:**

Spiders of the genus *Loxosceles* are distributed throughout tropical and temperate regions worldwide. *Loxosceles* spp. bites may evolve to necrosis, with or without intravascular hemolysis. There is no consensus regarding the best treatment to prevent necrosis. The objective of this study was to evaluate the factors associated with the development of necrosis and the impact that antivenom administration has on the evolution of cutaneous loxoscelism.

**Methodology/Principal findings:**

This was a prospective observational study carried out at a referral center for envenoming. Over a 6-year period, we included 146 patients with a presumptive or definitive diagnosis of loxoscelism. Depending on the symptom severity, a polyvalent anti-arachnid antivenom was administered or not—in 74 cases (50.7%) and 72 cases (49.3%), respectively. Cutaneous and systemic manifestations were assessed at admission and weekly thereafter. Adverse reactions to the antivenom were also evaluated. Cutaneous loxoscelism was observed in 141 cases (96.6%), and the spider was identified in 29 (19.9%). The mean time from bite to antivenom administration was 41.6 ± 27.4 h. After discharge, 130 patients (90.9%) were treated with corticosteroids, antihistamines and analgesics being prescribed as needed. The probability of developing necrosis was significantly lower among the patients who were admitted earlier, as well as among those who received antivenom (*p* = 0.0245). Among the 74 patients receiving antivenom, early and delayed adverse reactions occurred in seven (9.5%) and four (5.4%), respectively. Local infection was observed only in three (2.3%) of the 128 patients for whom that information was available.

**Conclusions/Significance:**

Necrosis after a *Loxosceles* sp. bite appears to more common when hospital admission is delayed or when antivenom is not administered. In addition, the administration of a polyvalent anti-arachnid antivenom appears to be safe, with a relatively low rate of adverse reactions.

## Author summary

Spiders of the genus *Loxosceles* are widely distributed around the world, and their bite may evolve to skin necrosis, a condition known as cutaneous loxoscelism. There is no consensus about the best treatment to avoid necrosis and ulceration. However, experimental studies suggest that the extent of the necrosis can be reduced by approximately 30% if antivenom is administered within the first 48 h after the venom injection. Therefore, we designed a study to follow patients diagnosed with loxoscelism, treated or not treated with antivenom, in order to assess the evolution of the cutaneous lesion. We observed that patients admitted earlier and receiving antivenom ≤ 48 h after the bite were less likely to develop necrosis, suggesting that antivenom administration has a protective effect, especially when administered promptly. Even the patients who received antivenom ≤ 60 h after the bite were less likely to develop a cutaneous ulcer.

## Introduction

Spiders of the genus *Loxosceles*, known as brown spiders or violin spiders, are widely distributed throughout the tropical and temperate regions of the world [1]. Envenoming caused by the bite of such a spider, referred to as loxoscelism, has mainly been reported in South America, the United States, and the Mediterranean region [2–7]. Loxoscelism can manifest as a cutaneous lesion at the site of the bite, which may evolve to skin necrosis and, more rarely, to intravascular hemolysis [5].

The venom of a *Loxosceles* spider is a complex mixture of proteins and peptide toxins. The main component of the venom, a phospholipase D toxin, also known as a sphingomyelinase D toxin, is responsible for inducing the skin necrosis and hemolysis observed in some cases [8,9]. Experimental studies have shown that the evolution to skin necrosis in loxoscelism is associated with the amount of venom injected [10,11], as well as with the species, sex, and ontogenetic stage of the spider involved [12–14].

Treatments described for the management of cutaneous loxoscelism vary depending on the stage of the lesion and the region of the world. Such treatments include antivenom, corticosteroids, dapsone, antihistamines, antibiotics, hyperbaric oxygen therapy, electric shock therapy, surgical excision, and vacuum-assisted wound closure [15,16]. However, because there is a lack of clinical studies, evidence for the use of any of those treatments is missing. An experimental study in rabbits injected with *Loxosceles intermedia* venom and treated with specific antivenom showed that the necrotic lesion was approximately 90% smaller when the antivenom was administered within 6 h after venom injection [17]. Even when the antivenom was administered 48 h after venom injection, the necrotic lesion was approximately 30% smaller than that seen in untreated controls.

Equine *Loxosceles* antivenoms are produced in Argentina, Brazil, Mexico, and Peru; all are (Fab’)2 antivenoms, except a total IgG antivenom produced in Peru. In Brazil, a *Loxosceles* antivenom has been produced since the 1960s [18]. The Brazilian national guideline recommends the administration of antivenom in loxoscelism, if warranted by the severity of the condition, without defining the timing of its administration [19]. At the hospital where the present study was carried out, the use of antivenom in cutaneous loxoscelism is indicated if there is no evidence of necrosis at admission, as determined by the physician in attendance. In cases of cutaneous-hemolytic loxoscelism, the antivenom is administered if there are signs of hemolysis.

The main objective of this study was to evaluate the evolution of patients who were bitten by *Loxosceles* sp. spiders and followed at a referral center for venomous animal contact, comparing those who were and were not treated with antivenom. Secondary objectives were to evaluate the factors associated with the development of necrosis and to determine the impact that antivenom administration has on the evolution of cutaneous loxoscelism.

## Methods

### Ethics statement

The study was approved by the Research Ethics Committee of the Emilio Ribas Institute of Infectious Diseases (Reference no. 38048314.1.0000.5538) also located in the city of São Paulo. All participating patients gave written informed consent.

### Study design and patients

This was a prospective observational study carried out over a 6-year period at Vital Brazil Hospital (VBH), Instituto Butantan, in the city of São Paulo, Brazil. The hospital is a referral center with extensive experience in the management of envenoming. The management of cases treated at the VBH is based on the Brazilian national guidelines [19].

From November 2014 to November 2020, patients admitted to the VBH with a *Loxosceles* sp. bite were invited to participate in the study. Those with a presumptive or definitive diagnosis of cutaneous or cutaneous-hemolytic loxoscelism were included in the study.

### Definitions

Cutaneous loxoscelism is defined as a painful macula at the bite site, with a mixture of violaceous and pale areas, hereafter referred to as a “marbled macula”. In some cases, there can be induration, and the wound is often surrounded by an erythematous area, which is not attributable to any other dermatological condition, typically appearing within the first three days after the bite. In some patients, the lesion may progress to an area of necrosis, with a well-defined border of ulceration and a base containing granulation tissue [16,20,21]. In the present study, any spider brought in was identified by a specialist, which allowed the definitive diagnosis of loxoscelism to be made.

Cutaneous-hemolytic loxoscelism is defined as intravascular hemolysis associated with a cutaneous lesion. Intravascular hemolysis is suspected when jaundice is present and is confirmed on the basis of the following biochemical variables (diagnostic cutoff values): total bilirubin (> 1.2 mg/dl), indirect bilirubin (> 0.9 mg/dl), and lactate dehydrogenase (> 250 U/L).

### Procedures

For cutaneous loxoscelism, there is no consensus regarding the timing of the administration of antivenom to avoid the progression of the lesion to necrosis. Therefore, if there was no evidence of necrosis, as determined by the physician in attendance, the administration of antivenom was optional. In contrast, antivenom administration was mandatory for patients with cutaneous-hemolytic loxoscelism, regardless of the evolution of the skin lesion. Patients received a polyvalent anti-arachnid antivenom (for *Loxosceles* spp., *Phoneutria* spp., and *Tityus* spp.), designated *soro antiaracnídico* (SAA, anti-arachnid serum), which is a (Fab’)2 immunoglobulin produced by the Instituto Butantan. It is dispensed in 5-ml vials, each milliliter capable of neutralizing 15 minimum necrotizing doses of *L*. *gaucho* venom. Patients in whom SAA administration was indicated first received an antihistamine (diphenhydramine: 1 mg/kg for children and 50 mg for adults) and a corticosteroid (dexamethasone: 0.15 mg/kg for children and 6–10 mg for adults). According to the Brazilian national guideline [19], patients with cutaneous loxoscelism should receive 25 ml of SAA and those with cutaneous-hemolytic loxoscelism should receive 50 ml.

Laboratory tests were performed at admission, at the first week after discharge, and whenever considered necessary thereafter [21]. For patients diagnosed with cutaneous loxoscelism, prednisone (40–60 mg/day for 5–7 days) was prescribed at discharge, as were an analgesic and an antihistamine for those who had pain and those who had pruritus, respectively.

The cutaneous lesion was evaluated at admission and at weekly visits, being measured along its longest axes, and the rectangular area was calculated. In patients who presented with more than one bite, the sum of the areas was used.

Adverse events related to the use of SAA, such as early and delayed hypersensitivity reactions, were assessed. Early hypersensitivity was diagnosed when adverse events occurred during the infusion of SAA or within the first 24 h thereafter, such events including urticaria, facial flushing, itchy throat, hoarseness, laryngeal stridor, cough, bronchospasm, nausea/vomiting, abdominal colic, hypotension, shock, hyperthermia, tremors, and chills. Delayed hypersensitivity (serum sickness) was defined as manifestations such as fever, pruritus, exanthema, urticaria, arthralgia, arthritis, and lymph node enlargement, occurring 5–24 days after antivenom administration [22].

Using a standard follow-up form, we collected data related to the following: demographic characteristics; pathological history; clinical evolution from bite to hospital admission; treatments prior to admission; local and systemic manifestations of envenoming; laboratory test results; and adverse reactions to SAA. In addition, patients were asked to keep a diary, in which they noted the presence and duration of the systemic manifestations of loxoscelism, as well as symptoms related to delayed adverse reactions to SAA.

Because of the coronavirus disease 2019 pandemic, several post-discharge evaluations conducted after March 2020 were carried out by telephone, especially those conducted for the investigation of delayed reactions to antivenom therapy. Nevertheless, face-to-face evaluations were conducted when necessary.

### Statistical analysis

A database was built and statistical calculations were performed with Epi Info software, version 6.04c (CDC, Atlanta, GA, USA) and the Stata software package, version 10.0 (StataCorp, College Station, TX, USA). Continuous variables are expressed as mean and standard deviation or as median and interquartile range, whereas categorical variables are expressed as absolute and relative frequencies. Proportions were compared by using chi-square tests or Fisher’s exact tests, whereas means and medians were compared by using Student’s t-tests or Mann–Whitney rank sum tests, as appropriate. Values of p < 0.05 were considered statistically significant. To quantify the effect that antivenom administration had on the development of necrosis, we calculated odds ratios (ORs) and the respective 95% confidence intervals (95% CIs).

## Results

**Fig 1:**
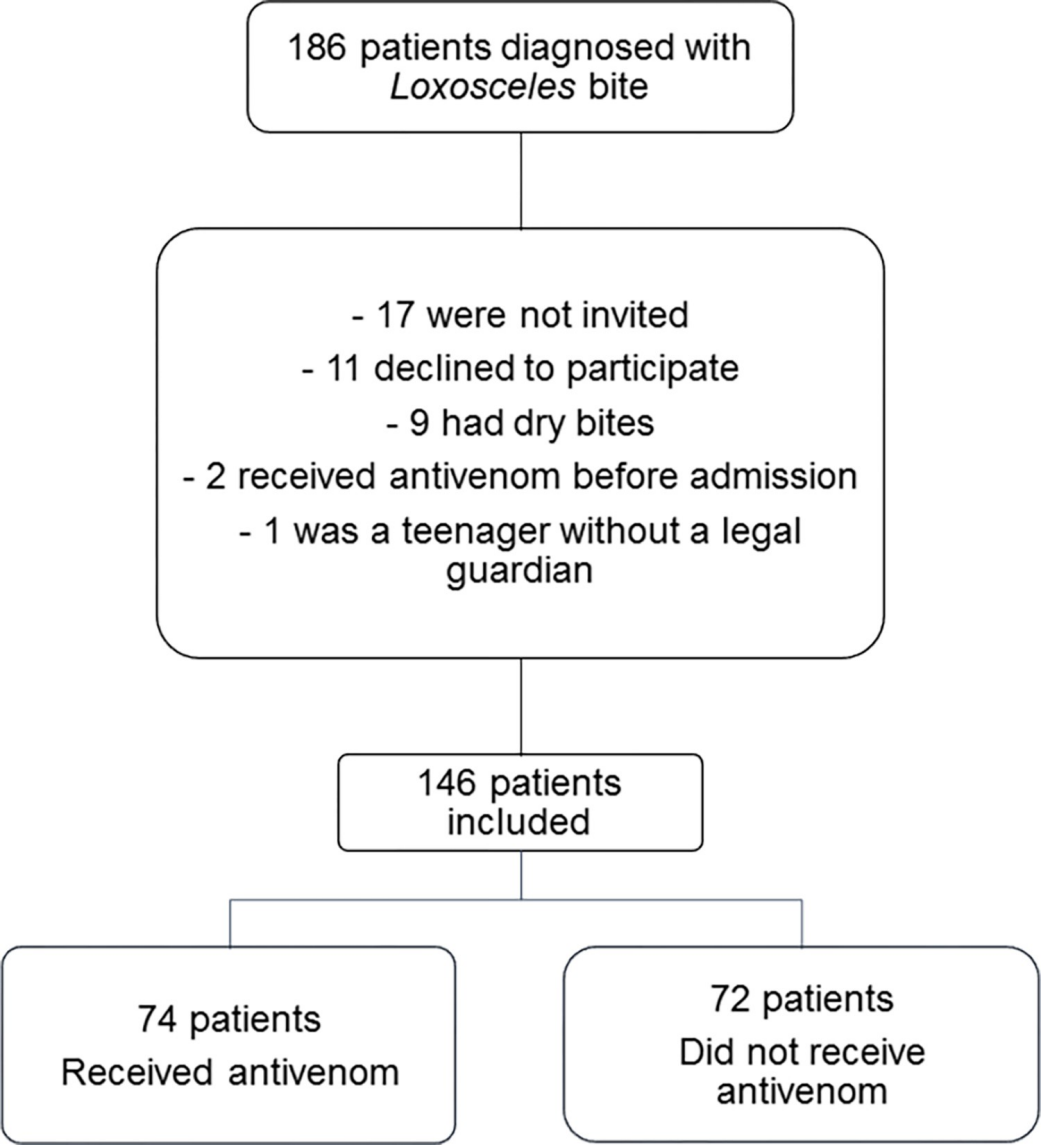
Study flow chart.

Between November 2014 and November 2020, 186 patients diagnosed with *Loxosceles* sp. spider bites were admitted to VBH. Of those 186 patients, 146 were included in the study (Fig 1).

### Admission

Of the 146 patients included, 124 reported the time of the bite. Among those patients, the mean time from bite to admission was 76.7 ± 78.0 h. Baseline demographic and clinical characteristics of the patients are shown in Table 1. Two of the patients were pregnant, and neither of those patients evolved to complications or adverse events, although only one received SAA.

**Table 1:** Baseline characteristics of patients with loxoscelism.

Characteristic	All patients	SAA		p
		Yes	No	
	(N = 146)	(n = 74)	(n = 72)	
Age (years), mean ± SD	41.3 ± 14.3	39.6 ± 14.7	43.0 ± 14.0	0.1711
Female, n (%)	72 (49.3)	40 (55.6)	32 (44.4)	0.2527
Hours from bite to admission, median (IQR)	58 (30.5–97)	33 (19–51)	96 (75–143)	< 0.0001
Preexisting conditions, n (%)				
Allergy	21 (14.6)	10 (13.7)	11 (15.5)	0.8162
Hypertension	11 (7.6)	7 (9.6)	4 (5.6)	0.5328
Hypothyroidism	10 (6.9)	4 (5.5)	6 (8.5)	0.5296
Diabetes mellitus	8 (5.6)	4 (5.5)	4 (5.6)	1.000
Current pregnancy	2 (1.4)	1 (1.4)	1 (1.4)	1.000
BMI (kg/m^2^), median (IQR)	26 (23.0–28.7)	26 (23.0–28.4)	26 (23.5–28.7)	0.5021

SAA: *soro antiaracnídico* (anti-arachnid serum); SD, standard deviation; IQR, interquartile range; BMI: body mass index.

The spider was observed by 72 (49.3%) of the 146 patients evaluated. In 29 (40.3%) of those 72 cases, the spider was identified: as *L*. *gaucho* in 21 (72.4%), as *Loxosceles* sp. in five (17.2%), and as *L*. *laeta* in three (10.3%).

**Fig 2:**
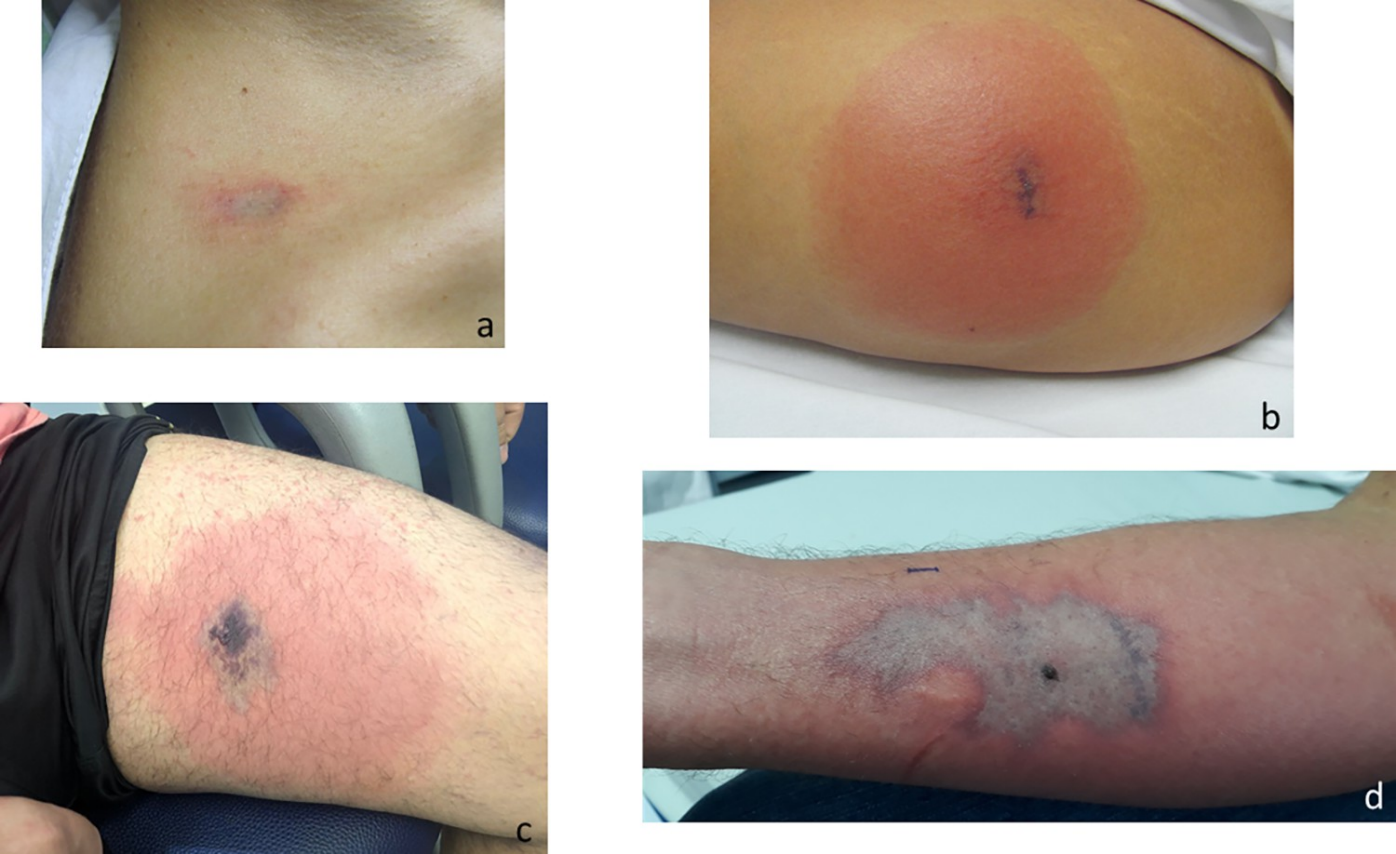
Marbled macula, with and without erythematous halo, after a *Loxosceles* sp. bite. (a) 18 h after the bite; (b) 24 h after the bite; (c) 72 h after the bite; (d) 120 h after the bite.

**Fig 3:**
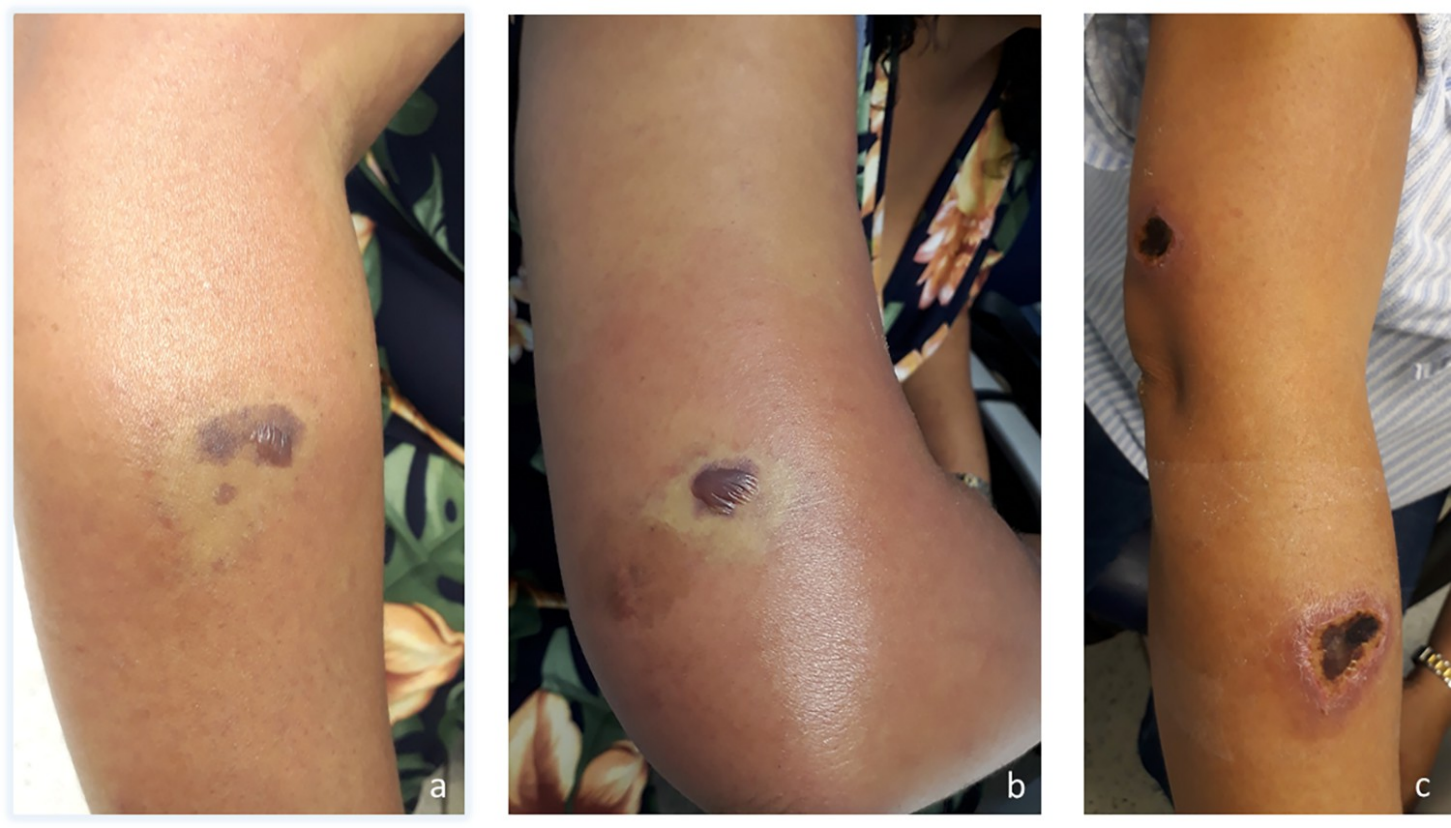
A patient bitten at two sites on the right arm. Marbled maculae on the forearm (a) and the upper arm (b), 4 days after a *Loxosceles* sp. bite. (c) Necrotic lesion on the upper arm and forearm 19 days after the bite.

**Fig 4:**
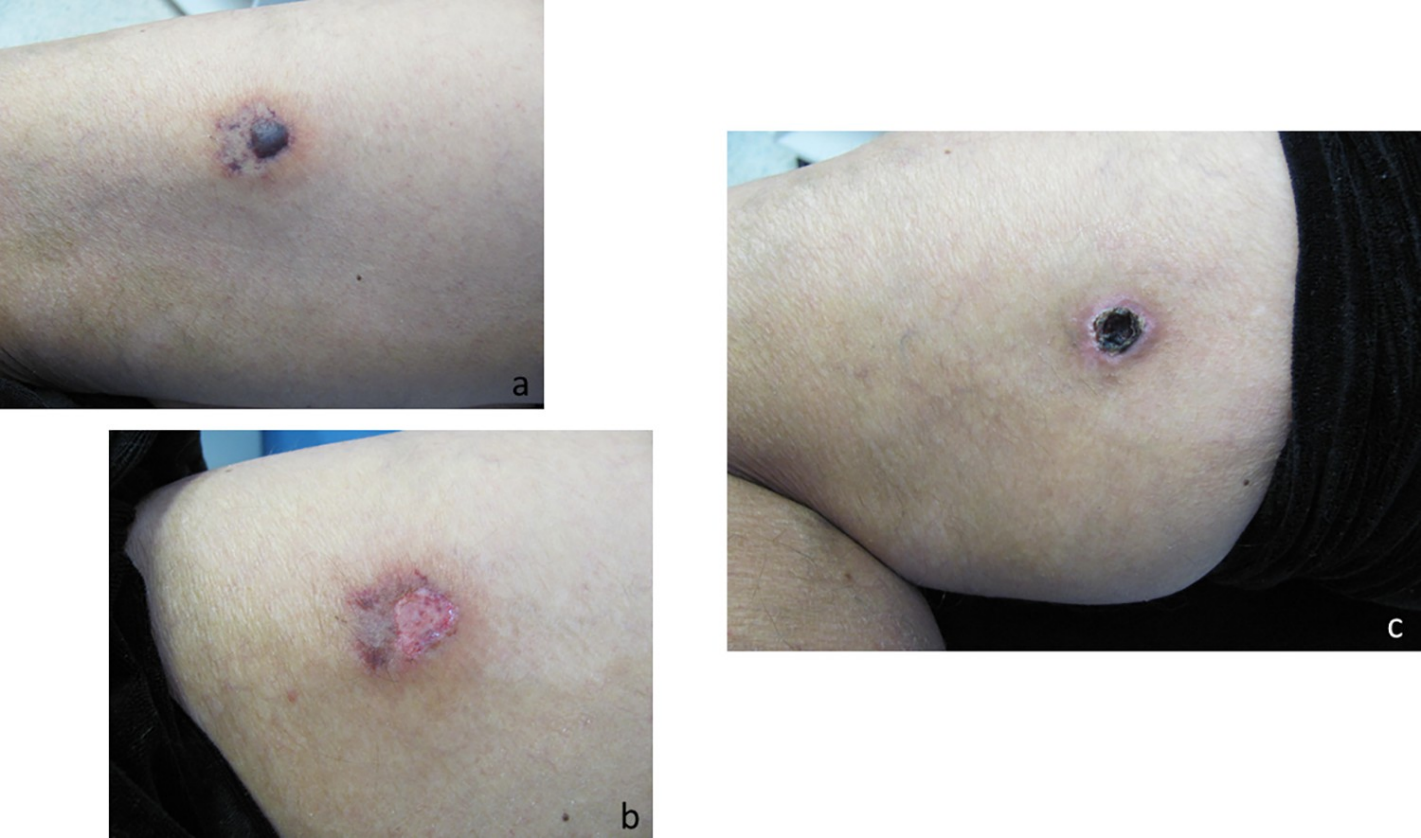
Evolution of a cutaneous lesion after a *Loxosceles* sp. bite. (a) marbled macula with blister, 7 days after the bite; (b) cutaneous lesion with a ruptured blister, 12 days after the bite; (c) eschar, 32 days after the bite.

**Fig 5:**
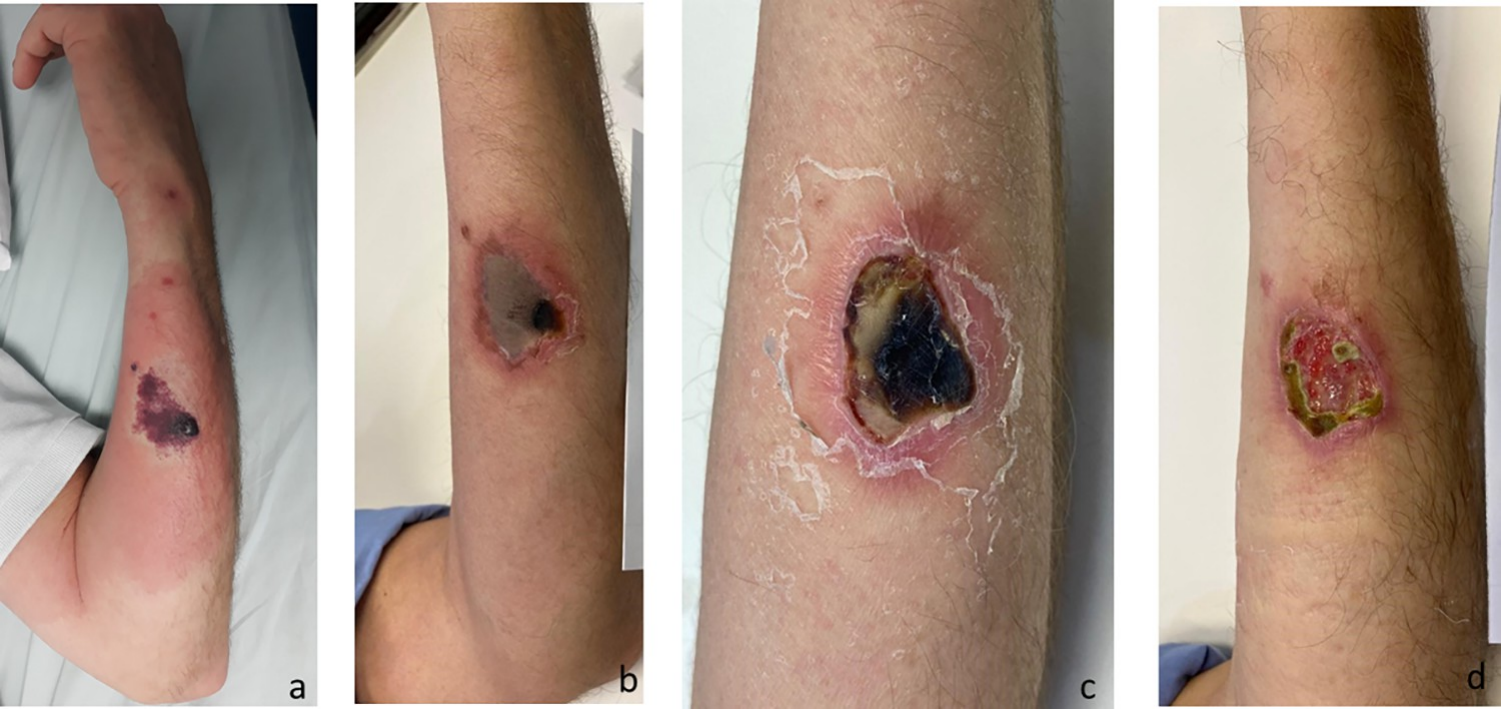
Evolution of a cutaneous lesion after a *Loxosceles* sp. bite, from marbled macula to necrosis and ulcer. (a) 4 days after the bite; (b) 17 days after the bite; (c) 24 days after the bite; (d) 31 days after the bite.

**Fig 6:**
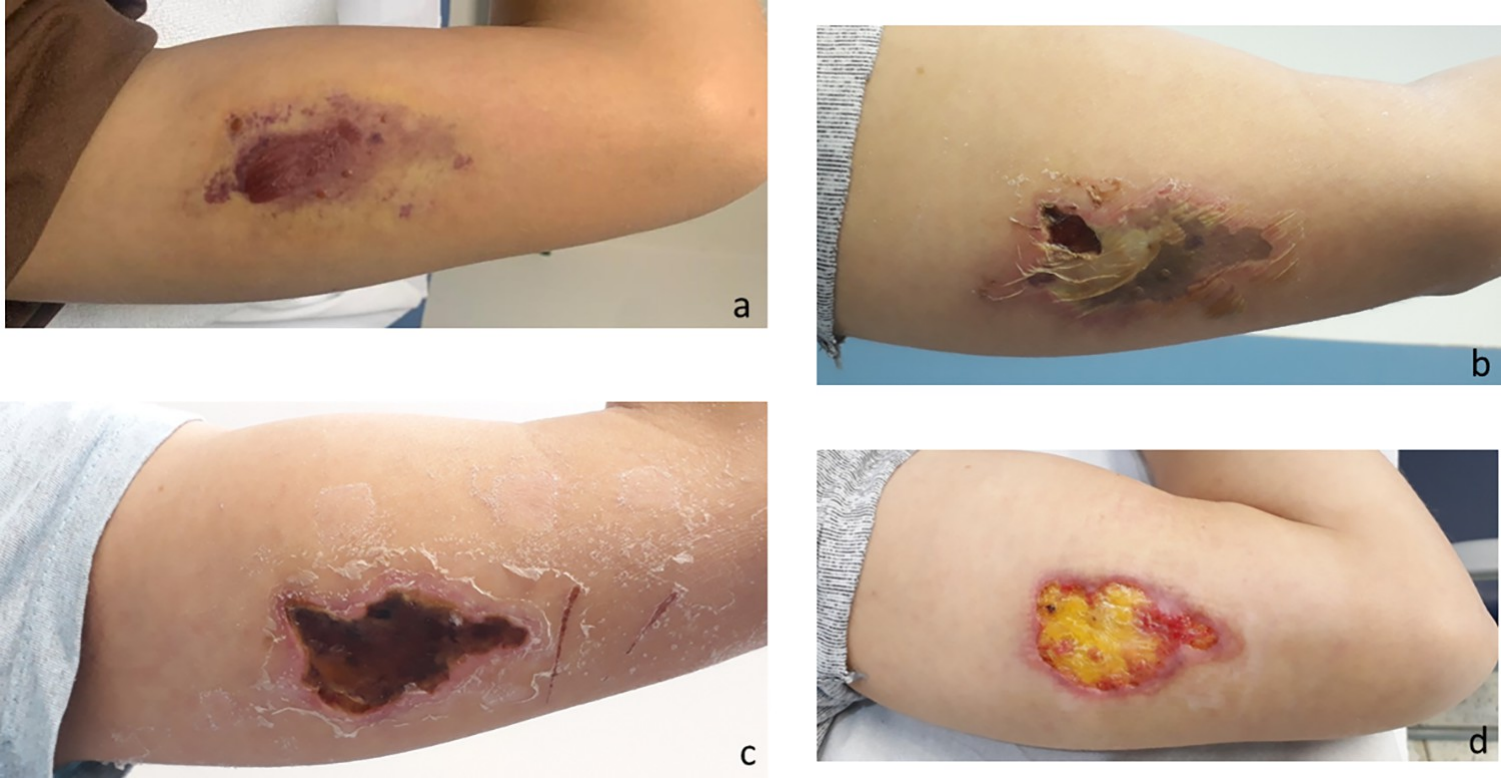
Evolution of the cutaneous lesion after a *Loxosceles* sp. bite in a patient with cutaneous-hemolytic loxoscelism. (a) 8 days after the bite; (b) 17 days after the bite; (c) 24 days after the bite; (d) 52 days after the bite.

**Fig 7:**
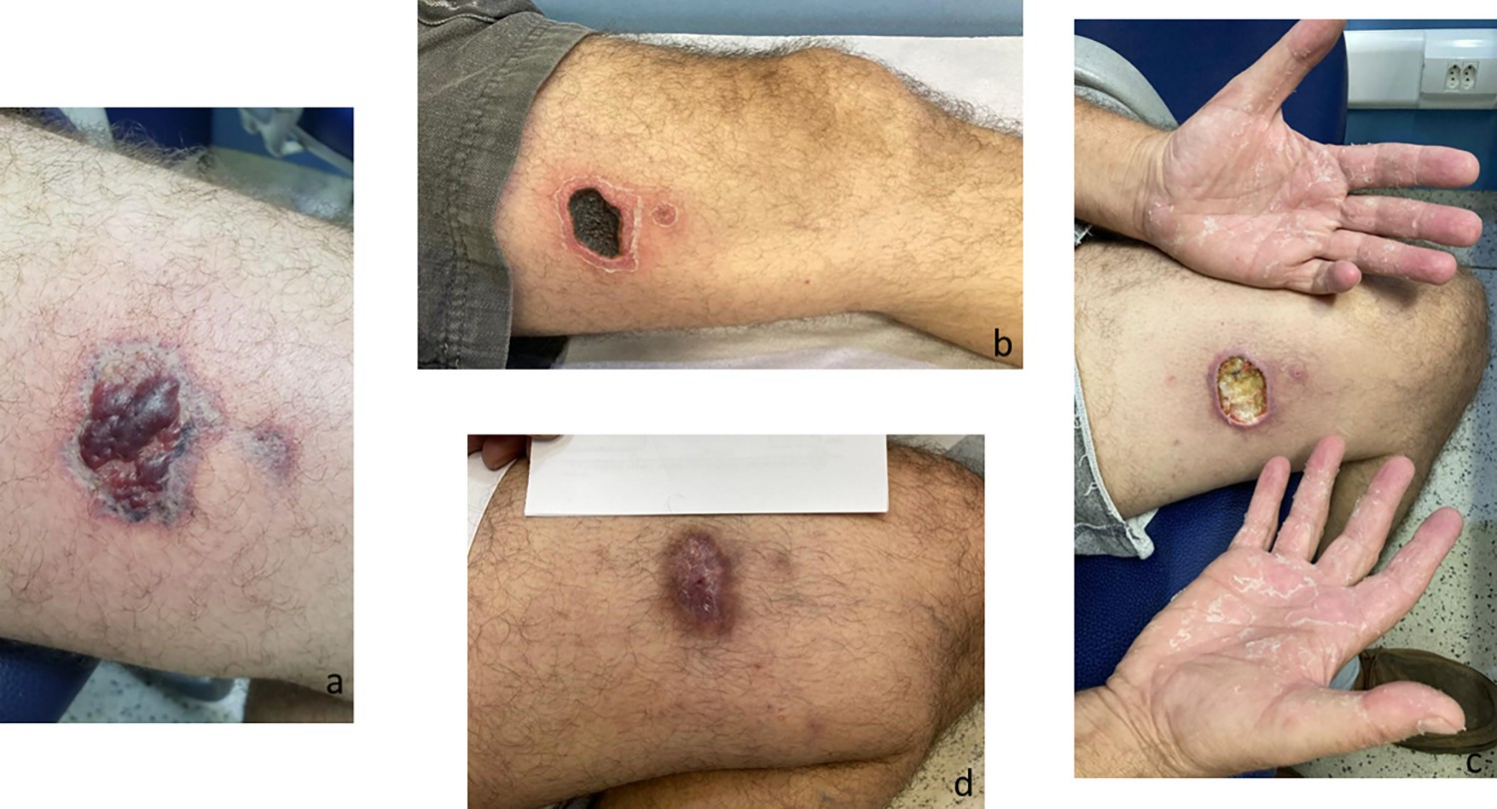
Evolution of a cutaneous lesion after a *Loxosceles* sp. bite, from marbled macula to necrosis and ulcer. (a) 5 days after the bite; (b) 20 days after the bite; (c) 27 days after the bite, the patient presented an ulcer at the bite site and scaling on the hands; (d) 83 days after the bite, the patient had a scar at the site of the bite but opted not to undergo plastic surgery.

**Fig 8:**
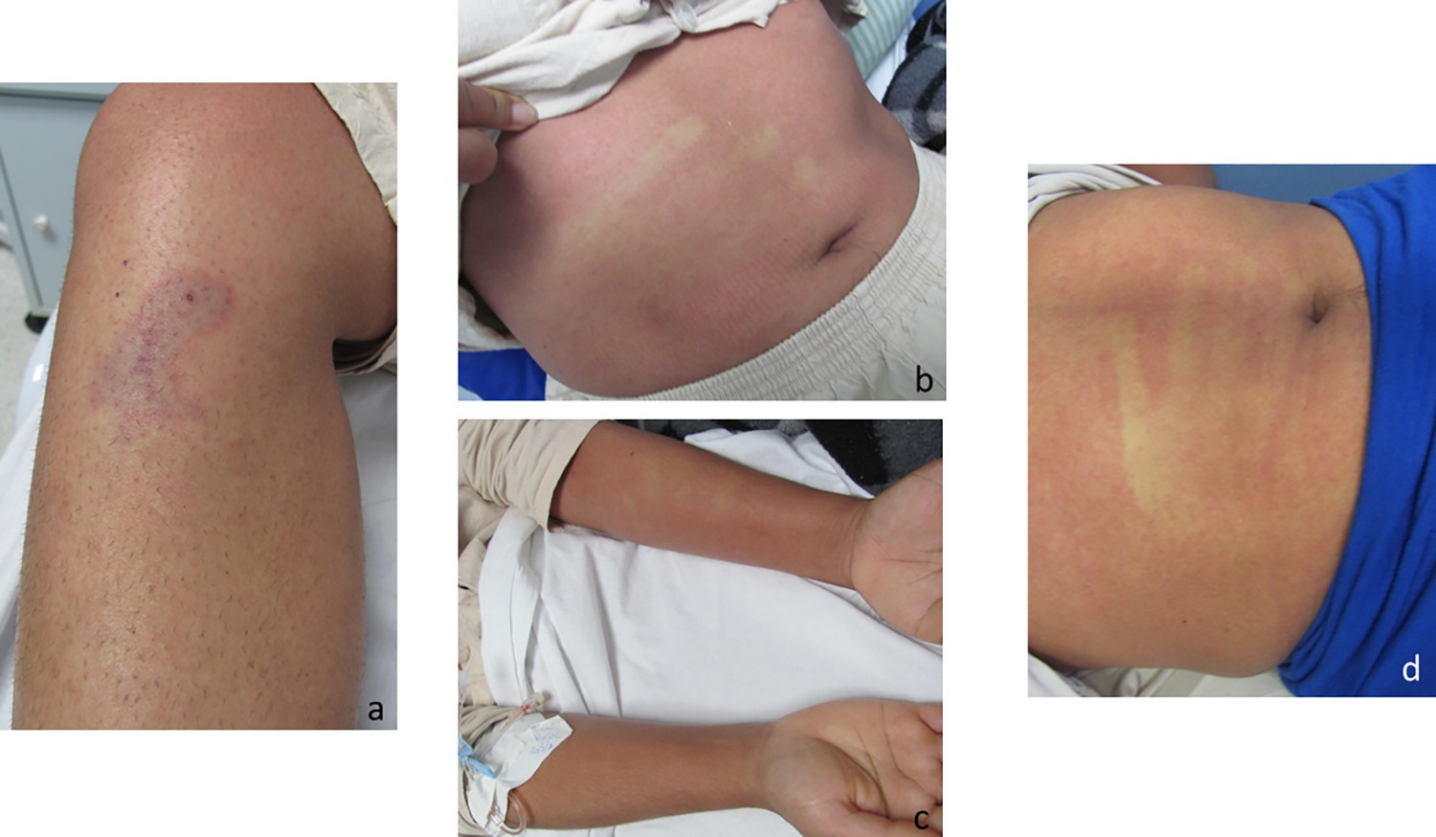
Evolution of a cutaneous lesion after a *Loxosceles* sp. bite. Marbled macula at the bite site (a), rash on the forearm (b), and rash on the abdomen (c), 4 days after the bite. (d) Persistence of the rash on the abdomen at 7 days after the bite.

Among the 146 cases evaluated, the main anatomical regions affected were as follows: the thigh, in 58 (39.9%); the trunk, in 37 (25.5%); the arm, in 18 (12.4%); the forearm, in 12 (8.3%); the leg, in 10 (6.9%); the face, in five (3.5%); and the neck, in three (2.1%). At admission, 15 (10.4%) of the patients had only erythema at the bite site, which did not evolve to a marbled macula. In those cases, the diagnosis was made by identifying the spider. Other patients presented with a marbled macula (Figs 2, 3A, 3B, 4A, 5A, 6A, 7A and 8A), induration, erythematous halo around the marbled macula (Figs 2B, 2C, 2D, 3B and 5A), necrosis (Figs 4C, 5B, 5C, 6B, 6C and 7D), and ulcers (Figs 5D, 6D, and 7C), which were seen in 114 (78.1%), 97 (67.8%), 98 (68.5%), 21 (14.4%), and two (1.4%), respectively. In 116 cases (82.3%), the patients presented with pain, which was categorized as mild in 31 (31.3%), moderate in 39 (39.4%), and severe in 29 (29.3%). Blisters were observed (at admission or thereafter) in 36 (24.8%) of the 146 cases (Figs 3A, 3B, 4A, 5A, 6A and 7A). Table 2 shows the systemic manifestations at admission, at least one of which was reported in 131 cases (89.7%).

**Table 2:** Systemic manifestations observed at hospital admission.

Clinical manifestation	n (%)
Exanthema*	108 (74.5)
Pruritus*	98 (67.6)
Malaise*	74 (51.0)
Erythema of the feet or hands*	62 (42.8)
Fatigue*	51 (34.9)
Headache*	52 (35.6)
Fever*	47 (32.4)
Nausea*	48 (33.3)
Jaundice	5 (3.4)

*Data available for only 145 of the 146 patients in the sample.

Of the 146 patients in the sample, 97 (66.4%) reported having received some treatment prior to admission, including antihistamines, in 56 (40.9%), corticosteroids, in 52 (37.9%), and antibiotics, in 28 (20.1%). All antibiotic therapy was discontinued at hospital admission. At admission, 141 (96.6%) of the patients were classified as having cutaneous loxoscelism and five (3.4%) were classified as having cutaneous-hemolytic loxoscelism.

### Treatment after hospital admission

Of the 146 patients evaluated, 74 (50.7%) received SAA. The mean and median times from bite to SAA administration were 41.6 ± 27.4 h and 36.0 h (22.0–55.5 h), respectively. The mean and median SAA administration times were 76.8 ± 30.9 min and 60 min (60–90 min), respectively. In 69 patients (93%), 5 vials of SAA were administered, and 5 patients (6.9%) received 10 vials of SAA. Age, sex, and the presence of comorbidities did not differ significantly between the patients who received SAA and those who did not. The frequency of SAA administration was higher among the patients who were admitted earlier (Table 1). Adverse reactions during SAA administration were observed in seven patients (9.5%), as follows: urticaria, in six (86%); facial flushing, in two (29%); cough, in one (14%); itchy throat, in one (14%); and nausea, in one (14%). In four (5.4%) of the 74 patients who received SAA, there was a delayed hypersensitivity reaction (serum sickness), which occurred 5–22 days after SAA administration. Although none of those patients presented all of the symptoms of serum sickness syndrome, one developed arthritis; one developed edema and erythema of the lips and eyes; one developed urticaria with pruritus and arthritis; and one developed disseminated pruritus.

At hospital discharge, prednisone was prescribed for 134 (91.8%) of the 146 patients. The mean and median times on corticosteroid therapy were 5.9 ± 1.9 days and 6 days (5–7 days), respectively, at mean and median doses of 0.6 ± 0.2 mg/kg and 0.6 mg/kg (0.5–0.7 mg/kg), respectively.

**Fig 9:**
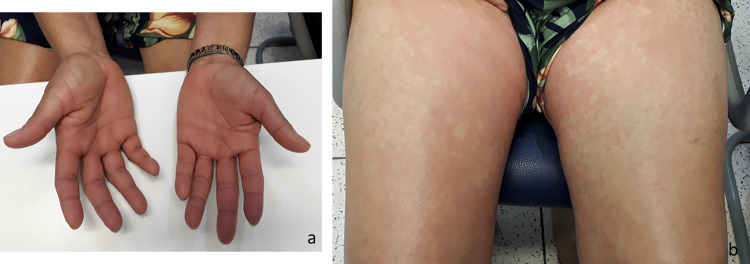
Rash on the thighs and palmar erythema in a patient bitten by a *Loxosceles* sp. spider, 4 days after the bite.

**Fig 10:**
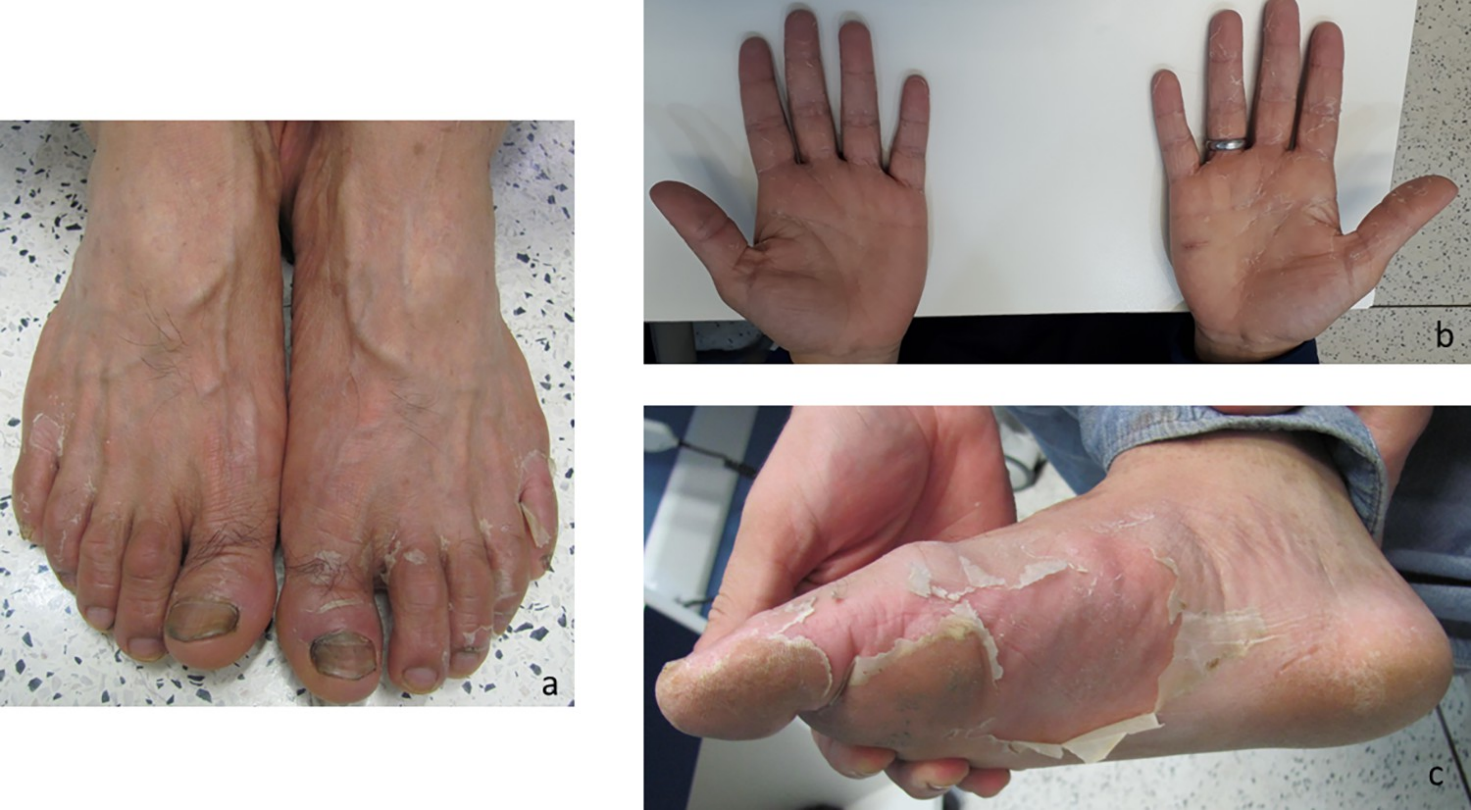
Patients bitten by a *Loxosceles* sp. spider and presenting desquamation on the feet and hands. (a) 17 days after the bite; (b) 24 days after the bite; (c) 36 days after the bite.

The duration of the systemic manifestations of loxoscelism varied considerably. As can be seen in Table 3, the systemic manifestations that were the most persistent were exanthema (Figs 8B, 8C and 9B), pruritus, and erythema of the feet or hands (Fig 9A). The duration of the other systemic manifestations was similar between the patients who received SAA and those who did not, with the exception of nausea, which lasted significantly longer among those in the latter group. Over the course of the follow-up period, cutaneous desquamation was observed in 50 (42%) of the 119 patients for whom that information was available. In 40 (80%) of those patients, the desquamation occurred on the hand (Figs 7C and 10C) or the foot (Fig 10A and 10B).

**Table 3:** Duration of systemic manifestations.

Manifestation	n	Duration (days)			p
		All patients	SAA		
			Yes	No	
		Median (IQR; range)	Median (IQR; range)	Median (IQR; range)	
Exanthema	81	7 (3–9; 1–42)	7 (3–10; 1–23)	7 (3–9; 1–42)	0.9346
Pruritus	77	7 (3–10; 1–36)	6 (2–11; 1–22)	7 (3–9; 1–36)	0.8376
Malaise	62	2 (1–7; 1–20)	2(1–5; 1–18)	2(1–9; 1–20)	0.3554
Erythema of the feet or hands	51	3 (2–9; 1–33)	5 (3–11; 1–24)	2 (1–7; 1–33)	0.0523
Tiredness	37	3 (2–8; 1–20)	2 (1–5; 1–13)	4 (2–10; 1–20)	0.1987
Headache	39	2 (1–4; 1–23)	2(1–3; 1–23)	2 (1–5; 1–20)	0.8950
Fever	35	1 (1–1; 1–3)	1 (1–1; 1–3)	1 (1–1; 1–3)	0.4183
Nausea	37	1 (1–4; 1–20)	1 (1–2; 1–8)	2 (1–7; 1–20)	0.0168

SAA: *soro antiaracnídico* (anti-arachnid serum); IQR, interquartile range.

There were 21 patients who already had necrosis or ulceration at admission. Of the remaining patients, 72 (62.1%) evolved to necrosis, the area of which ranged from 0.01 cm^2^ to 60 cm^2^. Among the patients questioned about the characteristic, ulcers were reported by 33 (29.5%), and the ulcerated area ranged from 0.08 cm^2^ to 64 cm^2^. Secondary infection was diagnosed in three (2.3%) of the 128 patients for whom that information was available, and the infection was diagnosed 13–28 days after the bite, after an eschar had formed.

When we excluded patients admitted with necrosis or ulcer, we found that evolution to skin necrosis was not associated with age (p = 0.1522), sex (p = 0.0545), body mass index (p = 0.4284), diabetes mellitus (p = 0.6482), hypertension (p = 0.7078), previous antihistamine use (p = 1.0000), previous corticosteroid use (p = 0.3136), total time of corticosteroid use (p = 0.42831), prednisone dose (p = 0.7660), or the presence of a systemic manifestation (p = 0.1757). The time from bite to admission was longer among the patients who evolved to necrosis, as was the time from bite to SAA administration and the time from admission to discharge. In addition, the area of induration and marbled macula at admission was larger among the patients who evolved to necrosis (Table 4). On the other hand, in the sample as a whole, male patients were more likely to develop necrosis. However, there was no sex difference regarding the mean and median times from bite to admission, which were 79.9 ± 100.4 h and 50.5 h (25.0–90.0 h), respectively, for females, compared with 73.4 ± 46.5 h and 68.0 h (38.0–101.0 h), respectively, for males (p = 0.1724). The same was true for the mean and median times from bite to SAA administration, which were 37.7 ± 27.3 h and 35.5 h (16.0–52.5 h), respectively, for females, compared with 46.5 ± 27.2 h and 42.0 h (26.5–57.5 h), respectively, for males (p = 0.1195). The antivenom was administered in 40 (55.6%) of the 72 female patients and in 34 (46.0%) of the 74 male patients (OR: 0.7; 95% CI: 0.4–1.3; p = 0.2527). However, at admission, the mean and median areas of marbled macula were larger in the male patients than in the female patients—21.1 ± 36.7 cm^2^ and 10.5 cm^2^ (3.0–23.0 cm^2^), respectively, vs. 11.0 ± 18.3 cm^2^ and 4.3 cm^2^ (1.5–11.3 cm^2^), respectively—and the difference was statistically significant (p = 0.0049).

**Table 4:** Comparison between patients who developed skin necrosis and those who did not.

Variable	Necrosis		OR (95% CI)	p
	Yes	No		
Age (years), mean ± SD	42.1 ±14.5	38.6 ± 14.9	–	0.1522
Sex, n (%)				
Female	33 (53.2)	29 (46.8)	2.3 (1.1–5.0)	0.0545
Male	39 (72.2)	15 (27.8)		
Hours from bite to admission, median (IQR)	60 (33–86)	40 (17–76)	–	0.0131
Hours from bite to SAA, median (IQR)	38 (26–59)	25 (11–42)	–	0.0106
Induration (cm^2^) at admission, median (IQR)	20 (10.5–27.0)	9 (1.5–18.0)	–	0.0012
Marbled macula (cm^2^) at admission, median (IQR)	7.0 (3.0–16.7)	2.9 (1.2–12.5)	–	0.0193
Days of corticosteroid use, median (IQR)	6.0 (5.0–7.0)	6.0 (5.0–6.5)	–	0.2831
Prednisone dose (mg/kg), median (IQR)	0.6 (0.5–0.7)	0.6 (0.5–0.7)	–	0.7660
Days from admission to discharge, median (IQR)	33 (28–52)	27 (15–32)	–	0.0000

OR: odds ratio; 95% CI: 95% confidence interval; SD, standard deviation; IQR: interquartile range; SAA: *soro antiaracnídico* (anti-arachnid serum).

Comparing the patients who received SAA (n = 65) with those who did not (n = 68), we found no significant difference in terms of the development of skin necrosis (61.5% vs. 72.1%). However, when we compared the patients who were admitted without skin necrosis ≤ 48 h after the bite and received SAA (n = 45) with those who were admitted > 48 h after the bite and did not receive SAA (n = 56), we found that the proportion of patients who developed necrosis was lower in the former group (51.1% vs. 73.2%), and the difference was statistically significant (Table 5). In addition, the proportion of patients who developed ulcers, which resulted from deeper necrosis, was lower among those who were admitted ≤ 60 h after the bite, had no necrosis at admission, and received SAA than among those who were admitted > 60 h after the bite and did not receive SAA—13 (24.1%) of 54 patients vs. 23 (48.9%) of 47 patients—and the difference was significant (OR: 0.3; 95% CI: 0.1–0.8; p = 0.0124).

**Table 5:** Frequency of cutaneous necrosis among patients who did and did not receive anti-arachnid serum, by the time from bite to antivenom administration.

	Time from bite to SAA administration	
	Any time	≤ 48 h
Necrosis with and without SAA, % vs. %	61.5 vs. 72.1	51.1 vs. 73.2
OR (95%CI)	0.6 (0.3–1.3)	0.4 (0.2–0.9)
p	0.2686	0.0245

At admission, the median lymphocyte count was lower among the patients who subsequently received SAA than among those who did not—1,342 cells/μl (764–485 cells/μl) vs. 1,737 cells/μl (1,206–2,337 cells/μl); p = 0.009—as were the median eosinophil count—176 cells/μl (56–403 cells/μl) vs. 330 cells/μl (158–513 cells/μl); p = 0.0152—and the median reticulocyte count—51,100 cells/μl (39,600–66,000 cells/μl) vs. 62,000 cells/μl (50,800–71,900 cells/μl); p = 0.0181. However, the median D-dimer level was higher in the former group—681 ng/ml (476–1,286 ng/ml) vs. 492 ng/ml (375–728 ng/ml); p = 0.0026—as was the median C-reactive protein level—23 mg/L (14–57 mg/L) vs. 15 mg/L (8.5–38.5 mg/L); p = 0.0377.

## Discussion

A bite from a *Loxosceles* sp. spider may progress to a necrotic cutaneous lesion. However, there is no clinical evidence to support the use of any specific treatment as being the most beneficial to prevent the progression to skin necrosis or to reduce the size of the lesion. In South America, some countries produce and use an anti-*Loxosceles* antivenom. In the present study, we evaluated 146 patients diagnosed with loxoscelism and found that those who received antivenom within the first 48 h after the bite were less likely to develop necrosis.

Approximately 80% of our patients were admitted with a marbled macula, and 10% had only erythema at the site of the bite, which did not evolve to a marbled macula. In the latter group, the diagnosis was confirmed by identifying the spider responsible for the bite. In our sample, only 20% of the patients captured the spider for identification. Other clinical studies have also reported a low frequency of spider identification [23,24]. Therefore, in most cases, the diagnosis of loxoscelism is based exclusively on the history, features of the lesion, and the presence of systemic manifestations. Patients were included in our study sample if they presented with a lesion that was clinically characteristic of a *Loxosceles* sp. spider bite and followed the typical clinical course.

More than 60% of the patients in our sample evolved to some degree of necrosis, including punctiform necrosis. In various studies of the effects of spider venom, necrosis has been reported to be quite common and has been managed with a variety of treatments, including antimicrobials and corticosteroids. Sezerino et al. [24] reported that more than 50% of the patients evaluated evolved to necrosis. Sams et al. [25] reported that 11 (58%) of 19 patients diagnosed with loxoscelism evolved to necrosis, and Del Puerto et al. [26] reported that 10 (59%) of 17 such patients evolved to residual ulcer. In the present study, with the exception of the spider species, mostly identified as *L*. *gaucho*, it was not possible to assess any of the other factor that have been found to be associated with necrosis in previous experimental studies [10–14]. However, the time from bite to admission was longer among the patients who evolved to necrosis than among those who did not.

In our study sample, the patients who received SAA ≤ 48 h after the bite were less likely to develop necrosis. Pauli et al. [17] studied the effect of antivenom therapy in rabbits injected with *Loxosceles* venom. The authors observed that even when the antivenom was injected 48 h after envenoming, the necrotic lesion was approximately 30% smaller in the animals that received antivenom than in those that did not. Their findings are in agreement with those of the present study, in which we showed that the administration of antivenom protected patients from developing necrosis. We found that even patients who received SAA ≤ 60 h after the bite benefited, because they were less likely to develop ulcers, which suggests that the necrosis was less extensive and more superficial, therefore being less prone to ulceration.

Early adverse reactions are a problem associated with antivenom administration. In the present study, the rates of early and delayed reactions to the administration of SAA, a polyvalent antivenom, were 9.5% and 5.4%, respectively. However, early reactions were mostly mild and not life-threatening. There were no anaphylactic reactions during the infusion of SAA, nor were there any subsequent signs or symptoms of severe serum sickness. There have been few studies describing the safety of anti-arachnid antivenoms. Sezerino et al. [24] reported that 6.5% of patients had an early reaction to the administration of specific anti-*Loxosceles* antivenom or SAA, although the authors provided no information regarding serum sickness. Nordt et al. [27] reported a 4% rate of adverse effects after the use of black widow antivenom. Isbister et al. [28] rates of 3.6% for early hypersensitivity reaction and 9% for serum sickness in patients who received equine (Fab’)2 antivenom against the *Latrodectus hasselti* spider species. A previous study carried out at our facility showed that 20% of patients who received *Loxosceles*-specific or polyvalent antivenom (SAA) had an early reaction [29]. The low frequency of early reactions observed in the present study might be attributable to the implementation of good antivenom production practices.

Despite the low frequency of adverse reactions to the polyvalent antivenom used in our study (SAA), we believe that there is no justification for maintaining the production of this trivalent antivenom (for *Loxosceles* spp., *Phoneutria* spp., and *Tityus* spp.). Given that the clinical manifestation of *Loxosceles* envenoming is totally different than that of envenoming caused by the other two genera, there is no need for a differential diagnosis among envenoming by a scorpion of the genus *Tityus*, a spider of the genus *Phoneutria*, and a spider of the genus *Loxosceles*. Therefore, we suggest that a *Loxosceles*-specific antivenom be produced.

In the present study, nearly all of the systemic manifestations of envenoming had a similar duration between the patients who received SAA and those who did not, the exception being nausea, which lasted longer in the latter. The most common systemic manifestations were exanthema and pruritus, both of which were observed in approximately 90% of the cases and lasted for approximately 6 weeks. In the patients who developed exanthema or pruritus, antihistamines were needed in order to control the symptoms. In loxoscelism attributed to various *Loxosceles* species, exanthema has been reported to occur in 22–100% of cases [5,7,23,30]. A translationally controlled tumor protein, also known as a histamine-releasing factor, has been described in *Loxosceles* spp. venoms [31]. A recombinant translationally controlled tumor protein of *L*. *intermedia* has been shown to cause edema and increase vascular permeability in mice [32].

In addition to the antivenom, our patients received corticosteroids, as recommended in the Brazilian national guideline [19]. Although there have been no experimental studies supporting the use of corticosteroids in cases of cutaneous loxoscelism [33,34], they are widely used in such cases [3,24]. In the present study, the patients who did and did not present with necrosis or ulceration received corticosteroids within a similar time window and at similar doses. The results of previous experimental studies, like those of the present study, indicate that it is important to review the approach to the use of corticosteroids in all patients with cutaneous loxoscelism.

Antimicrobials are frequently prescribed in cases of suspected loxoscelism [3,4,23,25,35]. In the present study, 20% of the patients were admitted with a prescription for an antimicrobial, which was suspended after loxoscelism was diagnosed. In our study sample, the prevalence of infection during follow-up was low (≈ 2%) and infection occurred after an eschar had formed. The differential diagnosis of cutaneous loxoscelism should include soft tissue infection. Because there are as yet no standardized tests for diagnosing loxoscelism, it is important to know the mechanism of action of the venom, as well as to monitor the clinical evolution of the skin lesion, in order to make an accurate diagnosis and prescribe the most appropriate treatment. Many skin conditions can be misdiagnosed as *Loxosceles* sp. bites, and we should avoid the temptation to “create zebras by painting stripes on horses” [36,37].

Of the two pregnant patients in our sample, one received SAA and had no adverse reaction. Neither of those patients evolved to complications or adverse events. Similarly, Anderson [38] evaluated five cases of loxoscelism in pregnant patients and reported a favorable evolution for the mother and baby in all five cases.

Our study has some limitations. Despite the prospective study design, data related to some variables were unavailable. Because few of the patients were admitted early, it was not possible to assess the effectiveness of antivenom administration at an earlier time point in a significant number of individuals. Despite being a prospective study, information was lost regarding the presence or absence of some variables, compromising a more accurate analysis. In addition, we were unable to assess the volume of each lesion. Although the lesions were heterogeneous, the area measurement was performed equally in patients who did and did not receive SAA. Furthermore, it was not possible to assess the depth of each lesion, even in the ulcer phase. Moreover, there is as yet no method to measure the amount of venom injected, which precluded any comparisons on that basis.

In conclusion, the likelihood of developing necrosis appears to be lower when patients with loxoscelism are admitted earlier and receive antivenom, suggesting that antivenom has a protective effect, especially if administered within the first 48 h after the bite, although it seems to be effective in preventing cutaneous ulcers even if administered within the first 60 h. In addition, treatment with the antivenom employed in the present study appears to be safe, with a relatively low rate of adverse reactions.
